# Geographic Representativeness of a Web-Based Smoking Cessation Intervention: Reach Equity Analysis

**DOI:** 10.2196/11668

**Published:** 2018-10-24

**Authors:** Michael S Amato, Amanda L Graham

**Affiliations:** 1 Schroeder Institute Truth Initiative Washington, DC United States; 2 Department of Oncology Georgetown University Medical Center / Cancer Prevention and Control Program Lombardi Comprehensive Cancer Center Washington, DC United States

**Keywords:** smoking cessation, health behavior, internet, population health, rural health, urban health, health equity, telemedicine

## Abstract

**Background:**

Cigarette smoking is the leading cause of preventable death and disease in the United States. Smoking prevalence is higher in rural areas than in metropolitan areas, due partly to differences in access to cessation treatment. With internet use at 89% of all US adults, digital approaches could increase use of cessation treatment and reduce smoking.

**Objective:**

We investigated the extent to which smokers from rural areas use a digital cessation resource. We compared the geographic distribution of registered users of a free Web-based smoking cessation program with the geographic distribution of US smokers.

**Methods:**

We mapped user-provided ZIP codes to Rural-Urban Continuum Codes. A total of 59,050 of 118,574 users (49.80%) provided valid ZIP codes from 2013 to 2017. We used US National Survey of Drug Use and Health data from 2013 to 2017 to compare the geographic distribution of our sample of Web-based cessation users with the geographic distribution of US smokers. Reach ratios and 95% confidence intervals quantified the extent to which rural smokers’ representation in the sample was proportionate to their representation in the national smoking population. Reach ratios less than 1 indicate underrepresentation.

**Results:**

Smokers from rural areas were significantly underrepresented in 2013 (reach ratio 0.89, 95% CI 0.87-0.91) and 2014 (reach ratio 0.89, 95% CI 0.86-0.92), proportionally represented in 2015 (reach ratio 1.08, 95% CI 1.02-1.14) and 2016 (reach ratio 1.03, 95% CI 0.94-1.14), and proportionally overrepresented in 2017 (reach ratio 1.16, 95% CI 1.12-1.21). Smokers from Large Metro areas were proportionally represented in 2013 and 2014 but underrepresented in 2015 (reach ratio 0.97, 95% CI 0.94-1.00), 2016 (reach ratio 0.89, 95% CI 0.85-0.94), and 2017 (reach ratio 0.89, 95% CI 0.86-0.91).

**Conclusions:**

Results suggest that smokers from rural areas are more than proportionally reached by a long-standing digital cessation intervention. The underrepresentation of smokers from Large Metro areas warrants further study.

## Introduction

### Geographic Disparities in Smoking Prevalence

The disease burden from cigarette smoking—still the leading cause of preventable death and disease in the United States [1]—disproportionately affects rural Americans [2-4]. Although the national prevalence of cigarette smoking was 13.9% in 2017, sharp geographic disparities exist: adults living outside metropolitan areas were nearly twice as likely to smoke as their urban counterparts (21.5% and 11.5%, respectively) [5]. While there has been a substantial decline in smoking prevalence in urban populations, smoking rates between 2007 and 2014 remained stagnant in rural areas [6]. Rural residence has been shown to be an independent predictor of this difference in smoking trends, even after controlling for important covariates [6-8]. Understanding this geographic disparity and reducing the higher rates of smoking among rural Americans has been acknowledged as a public health priority [9,10].

Rural populations and cultures are heterogeneous, as are the challenges they face when quitting tobacco [7,11]. Among these are barriers to treatment access, including lack of insurance [12], geographic isolation, and limited access to trained tobacco treatment providers [10]. The internet is often the first place that many smokers turn to for health information and is well suited to address barriers to cessation treatment access for rural adults. In 2017, 36% of all smokers in the United States—more than 12 million adults—searched online for information about quitting smoking [13]. Evidence-based internet interventions deliver the core components of cessation treatment through engaging, multimodal formats, often free of charge to the end user, and yield quit rates comparable with in-person and phone-based interventions [14,15]. They are available around the clock and (uniquely) can be accessed conveniently at times when smokers most need support to prevent relapse. Thriving, open access online social networks for smoking cessation (eg, BecomeAnEX [16]) provide connection to a broad range of current and former smokers for real-time information and support that may be lacking in rural smokers’ own social networks [17].

However, enthusiasm for the potential of internet interventions to reduce smoking prevalence among rural adults may need to be tempered by the realities of a persistent digital divide. According to the Pew Research Center, 89% of all American adults used the internet in 2018, yet disparities in usage rates remain [18]. For almost two decades, rural adults have been roughly 10 percentage points less likely to use the internet than their urban and suburban counterparts. In 2018, 78% of rural adults reported that they used the internet, compared with 92% of urban adults and 90% of suburban adults [18]. In addition to differences in access, differences in bandwidth may affect rural adults’ ability to take advantage of rich, interactive features: as of 2016, broadband was available to 96% of urban Americans but only 61% of rural Americans [19].

To our knowledge, only 1 study has examined the reach of internet smoking cessation interventions across the rural-urban continuum. In 2007, Danaher et al [20] compared the proportion of rural adults enrolled in a Web-based trial for smokeless tobacco cessation with the proportion of rural adults in the US national population. Using a chi-square test, they found that a significantly greater proportion of trial participants lived in rural areas (8.1%) than would be expected based on the national proportion of adults who lived in rural areas (4.2%). That result reflected both smokeless tobacco usage patterns and the success of the study’s targeted marketing efforts.

### Objectives

In this study, we extended the work of Danaher et al to investigate the participation of rural US smokers in a free, evidence-based, digital smoking cessation intervention. Instead of the chi-square statistic, we employed the reach ratio (ReRa) [21], a measure of population reach that has been used to document the extent to which specific subgroups of a population benefit from a public health intervention [21-23]. The benefit of the ReRa method is that it provides not only a significance test, but also an estimate of magnitude and confidence interval for numeric comparison. A ReRa of 1.0 indicates that a group is perfectly represented, greater than 1.0 indicates overrepresentation, and less than 1.0 indicates underrepresentation. For example, Baskerville et al [22] used ReRas to examine the equity of quitline treatment reach across 3 specific vulnerable populations (young males; those with less than high school education; and rural dwellers) following implementation of tobacco warning labels with the toll-free quitline number. They found substantial variability in the reach equity of quitlines for rural populations across Canadian provinces, ranging from approximately 0.1 for rural smokers in Manitoba to approximately 1.4 for rural smokers in New Brunswick. Similarly, Amato et al [23] examined quitline use in Minnesota and found that rural Minnesotans were less likely than their urban counterparts to use quitline services (ReRa 0.78). In this study, we used ReRas to measure the extent to which the proportion of smokers who registered for a free Web-based smoking cessation program from rural areas matched their representation in the national population.

## Methods

### Study Setting

We extracted data from 2013 to 2017 from BecomeAnEX, a free Web-based smoking cessation program developed by Truth Initiative (Washington, DC, USA) in collaboration with Mayo Clinic (Rochester, MN, USA). The program was launched in 2008 [24] and delivers cessation treatment consistent with US national treatment guidelines [25]. Smokers find BecomeAnEX through organic search, using common search phrases like “how to quit smoking.” Smokers also find BecomeAnEX through paid advertisements on Google search. Smokers anywhere in the United States had equal opportunity to register on BecomeAnEX (ie, no geotargeting of advertisements was in place during the study period). Upon registration, users provide an email address, choose a username, and designate a password. During the study period, users could also choose to provide optional pieces of personal information at registration, including ZIP code, age, gender, and smoking history.

Procedures for collecting ZIP code information on BecomeAnEX changed during the study period. During most years, ZIP code was a required element of the registration process; during 2015, 2016, and part of 2017, it was entered optionally. As a result, the proportions of users who entered a ZIP code during those years were substantially lower than during others. To ensure that estimates of reach were not driven by sample differences, we assessed the effects of gender and age on likelihood of reporting a valid ZIP code using separate quasi-Poisson logistic regressions. Age was entered as a continuous variable measured in decades; female gender was entered as an indicator variable. Interaction terms with year were entered as categoric variables.

### Geographic Classification of Users

We used ZIP codes from user registration data to calculate ReRas following 2 preprocessing steps. In the first preprocessing step, we classified BecomeAnEX users in terms of location. We mapped each user’s ZIP code to a 2013 US Department of Agriculture Rural-Urban Continuum Codes (RUCC) value. RUCC provides a classification system for distinguishing metropolitan counties based on the size of the metropolitan area, and for nonmetropolitan counties based on the extent of urbanization and proximity to metropolitan areas, drawing on the 2010 US Census and the 2006-10 American Community Survey [26]. We mapped ZIP codes to ZIP Code Tabulation Areas (ZCTAs), then ZCTA to county, and finally county to 2013 RUCC [27]. Demographic differences in the likelihood of providing a ZIP code during registration were assessed with logistic regression.

We selected the RUCC classification system to allow direct comparison with detailed tables from the US National Survey of Drug Use and Health (NSDUH), which also report geographic area using the RUCC system. Although RUCC uses a 9-point classification system, we combined geographic area types into the following 3 categories to ensure that samples in all groups were of sufficient size to support meaningful inference based on confidence intervals: Large Metro (RUCC 1), Small Metro (RUCC 2 or 3), and Nonmetro (RUCC 4-9) [27]. Large Metro areas had populations of 1 million or more people. Small Metro areas had populations of fewer than 1 million people but were defined as metropolitan areas based on population and worker commuting criteria by the US Office of Management and Budget. Briefly, those criteria included an urbanized core of 50,000 or more population and adjacent areas that were socially and economically integrated [28]. Nonmetro areas were counties outside of defined metropolitan areas [26]. Although our definition of Nonmetro was not strictly limited to individuals living in areas classified as “completely rural” (RUCC 9), it included the roughly 20% of US smokers living at the rural end of the rural-urban continuum. Previous research has also grouped geographic classifications [2-4,6,7,10].

### Calculation of Reach Ratios

In a second preprocessing step, we calculated numerators (proportions of BecomeAnEX users) and denominators (proportions of US smokers) for the ReRas. We defined numerators for ReRas as the proportions of BecomeAnEX users from each geographic category within each year. These proportions were calculated from the geotagged BecomeAnEX registration data. For example, in 2016, 43.8% of new users on BecomeAnEX lived in Large Metro areas (Table 1, bottom panel).

We defined denominators for ReRas as the proportions of US smokers from rural and urban areas. These proportions were calculated from NSDUH estimates of the numbers of smokers by geographic category (Table 2.56A for 2013 [29]; Table 2.56A for 2014 [30]; Table 2.41A for 2015 and 2016 [31]; and Table 2.41A in 2017 [32]). Proportions were calculated as the estimated number of smokers within each geographic category, divided by the total number of smokers. For example, in 2016 there were 51,333,000 smokers aged 12 years and over living in the United States, of whom 25,259,000 lived in Large Metro areas (49.2%; Table 1, top panel; Table 2.41 in 2016 NSDUH detailed tables [31]).

Finally, we created ReRas from the 2 sets of proportions. Following the approach of Campbell et al [21], we calculated 95% confidence intervals for each ReRa using the Wald interval method (section 4.2.1 in Fagerland et al [33]). The key question of interest was whether ReRas for each group significantly differed from 1.0 in any years.

**Table 1:** Proportions of smokers in the US national population and among users of a Web-based intervention, by location and year.

Population and type of area (RUCC^a^)		Year, %				
		2013	2014	2015	2016	2017
**US smokers^b^**						
	Large Metro (1)	48.14	50.07	50.29	49.21	48.18
	Small Metro (2,3)	31.63	31.00	32.93	31.99	33.65
	Nonmetro (4-9)	20.23	18.93	16.78	18.80	18.17
**Geographically classified BecomeAnEX users^c^**						
	Large Metro (1)	48.86	50.63	48.86	43.85	42.65
	Small Metro (2,3)	33.16	32.53	33.05	36.70	36.24
	Nonmetro (4-9)	17.98	16.83	18.08	19.45	21.11

^a^Rural-Urban Continuum Codes.

^b^Total numbers of smokers (in thousands) aged ≥12 years living in the United States were 55,778 in 2013; 55,240 in 2014; 51,951 in 2015; 51,333 in 2016; and 48,692 in 2017.

^c^Total numbers of BecomeAnEX users who reported a valid ZIP code were 33,484 in 2013; 18,255 in 2014; 5491 in 2015; 1820 in 2016; and 8832 in 2017.

## Results

### Sample Characteristics

Between 2013 and 2017, a total of 127,207 new users registered on BecomeAnEX. Among those users, 67,854 provided valid ZIP codes and were assigned a RUCC value, representing 53.34% of all new users during the time period. Virtually all (114,373/114,844, 99.95%) users were current smokers or former smokers. Age was provided by 77.22% (98,227/127,207) of users; gender was provided by 76.61% (97,449/127,207). For every additional decade of a user’s age at registration, they were on average 3% more likely to provide a ZIP code across all years in the study period (relative risk 1.03, 95% CI 1.027-1.031, ranging from 1.00 in 2016 to 1.03 in 2013). Women were 10% more likely than men to provide a ZIP code (relative risk 1.10, 95% CI 1.103-1.12, ranging from 1.01 in 2015 to 1.44 in 2016).

Table 1 shows the proportions of smokers in each geographic area by year, for both the national population and the treatment-seeking population of BecomeAnEX users. In both samples, the largest group in all years lived in Large Metro areas (43.85% to 50.63%), while the smallest group lived in Nonmetro areas (16.83% to 21.11%). About a third in both samples lived in Small Metro areas (31.00% to 36.70%).

Table 2 shows the extent to which the geographic distribution of smokers seeking treatment on BecomeAnEX mirrored the geographic distribution of smokers in the general population, with ReRas and 95% confidence intervals.

### Large Metro Areas

In 2013 and 2014, the proportions of BecomeAnEX users from Large Metro areas (RUCC=1) were similar to their representation in the general smoking population. Beginning in 2015, smokers from Large Metro areas were marginally underrepresented (ReRa 0.97, 95% CI 0.94-1.00), and in 2016 and 2017 they were underrepresented by an even greater extent (2016: ReRa 0.89, 95% CI 0.85-0.94; 2017: ReRa 0.89, 95% CI 0.86-0.91).

### Small Metro Areas

In contrast, smokers from Small Metro areas (RUCC 2 or 3) were overrepresented in all years except 2015. The magnitude of the disparity was greatest in 2016 (2013: ReRa 1.05, 95% CI 1.03-10.7; 2014: ReRa 1.05, 95% CI 1.02-1.08; 2016: ReRa 1.15, 95% CI 1.08-1.22; 2017: ReRa 1.08, 95% CI 1.05-1.11).

### Nonmetro Areas

The representation of Nonmetro smokers (RUCC 4-9) on BecomeAnEX increased the most during the study period. They were underrepresented in 2013 (ReRa 0.89, 95% CI 0.87-0.91) and 2014 (ReRa 0.89, 95% CI 0.86-0.92), overrepresented in 2015 (ReRa 1.08, 95% CI 1.02-1.14), proportionally represented in 2016 (ReRa 1.03, 95% CI 0.94-1.14), and then overrepresented again in 2017 (ReRa 1.16, 95% CI 1.12-1.21).

**Table 2:** Reach ratios (ReRas) and 95% confidence intervals for the geographic distribution of a Web-based smoking cessation intervention in the United States.

Type of area (RUCC^a^)	Year, ReRa (95% CI)				
	2013	2014	2015	2016	2017
Large Metro (1)	1.01 (1.00-1.03)	1.01 (0.99-1.03)	0.97 (0.94-1.00)	0.89 (0.85-0.94)^b^	0.89 (0.86-0.91)^b^
Small Metro (2,3)	1.05 (1.03-1.07)^b^	1.05 (1.02-1.08)^b^	1.00 (0.96-1.04)^b^	1.15 (1.08-1.22)^b^	1.08 (1.05-1.11)^b^
Nonmetro (4-9)	0.89 (0.87-0.91)^b^	0.89 (0.86-0.92)^b^	1.08 (1.02-1.14)^b^	1.03 (0.94-1.14)	1.16 (1.12-1.21)^b^

^a^Rural-Urban Continuum Codes.

^b^ReRa significantly different from 1.

## Discussion

### Principal Results

This study examined the extent to which registered users on a free Web-based smoking cessation program were geographically representative of the US national smoking population. Results indicate that the reach of BecomeAnEX was relatively similar across the rural-urban continuum. We observed some differences, although the magnitude of difference was relatively small when compared with other published ReRas [21,22]. The reach to rural areas was of particular interest, because smokers in those areas often have higher smoking rates and reduced access to other forms of cessation treatment.

Summarizing across all years, there is evidence for two general trends. First, despite the digital divide, smokers from Nonmetro areas appeared to seek out and use digital cessation resources at a rate that was proportional to—and even higher than—their representation in the population. The expansion of broadband availability and smartphone penetration may have facilitated the proportional increase from 2013 and 2014. Second, we observed a proportional decrease in BecomeAnEX users from Large Metro areas. One speculative hypothesis for that trend could be higher prevalence of nondaily and social smoking among smokers in Large Metro areas; many nondaily or social smokers do not view themselves as smokers and do not seek cessation treatment [34,35]. Alternatively, smokers in Large Metro areas might be using other types of cessation resources. Given the large proportion of the population living in Large Metro areas and the substantial population health benefits of quitting smoking, future research should explore this unexpected finding.

### Limitations

These analyses have several limitations. First, the geographic classification of BecomeAnEX users and NSDUH respondents was based on 2013 RUCC codes, which are updated every 10 years following each US Census. The census is the best available data for our purpose but does not capture changes that may have occurred between individual years in the study period. Second, other rural-urban classification systems with more nuanced categories may have provided additional insight [36]. We chose to use the RUCC system because it is widely used throughout the literature. We chose to combine all Nonmetro RUCC classifications into a single category due to sample size limitations. It is possible that smokers in the least connected areas may face unique challenges to quitting that were not detected in our analysis. However, analyzing them independently resulted in unacceptably large confidence intervals that prevented meaningful interpretation (not reported). Future research should investigate how smokers in the most rural communities access evidence-based cessation treatment, using alternative methods. Nonetheless, the reported ReRas for the combined category provides a useful and previously unavailable estimate of treatment reach for 20% of the smoking population. Third, although the method used to create confidence intervals has been previously published and is the best available to our knowledge, it ignores variability in NSDUH smoking prevalence estimates, while simultaneously overestimating error variance in the ReRas for each individual year by ignoring cross-year correlations. Analytic techniques that overcome these methodological limitations are needed, such as a method for multinomial trend analysis of ReRas.

Fourth, ZIP codes were available for only 53.34% of registered users. Although older users and women were more likely than younger users and men to provide ZIP codes, we have no reason to expect systematic differences by age or gender between rural and urban US adults in their proclivity to provide ZIP codes during the registration process but cannot rule out the possibility. Differences in rates of ZIP code missingness would not be expected to affect the robustness of our findings, unless there were an interactive effect of gender or age with geographic area type on a smoker’s likelihood of seeking treatment from an internet intervention. We are not aware of any data to suggest such an interaction exists. Previous research has found that the geographic disparity in smoking rates is greater for women than for men [8]. That finding does not suggest disparities in treatment seeking and does not directly affect our conclusions, but warrants further study. Future research, using appropriate population surveillance methods that can control for multiple covariates, should further investigate demographic correlates of geographic disparities.

Finally, our research focused on one specific Web-based cessation program, and therefore our results are not synonymous with the reach of all digital cessation interventions across the rural-urban continuum. However, our approach provides an easily replicable model for other intervention platforms to document their reach based on geography.

### Conclusions

Progress is needed in addressing other challenges facing rural communities. Comprehensive smoke-free air laws, changes in social norms around tobacco use, effective tobacco industry countermarketing, and limiting children’s access to tobacco products are among the changes that still need to reach many rural communities. However, with the broad (and increasing) reach and proven effectiveness of internet smoking cessation interventions, access to treatment should not remain a barrier to quitting among rural smokers.

## Abbreviations

NSDUH: National Survey of Drug Use and Health
ReRa: reach ratio
RUCC: Rural-Urban Continuum Codes
ZCTA: ZIP Code Tabulation Area
